# Extra-Limites

**Published:** 1842-10-01

**Authors:** 


### EXTRA-LIMITES.

*[To the following representations are due the recent improvements, ordered by Government, in the pensions, promotion, and discipline of the Medical Department of the Indian Army.]*

### Note on the Present Condition of the Medical Department of the Indian Army. By *J. R. Martin*, Esq. Surgeon on the Bengal Establishment.

In what is here submitted to Authority on the subject of the Medical Department of India, I shall beg leave first, to offer a few observations of a general nature ;—secondly, to state the results of the present mode of managing the affairs of the Services through boards composed of the three senior officers on the list, and who, in Bengal at least, rise by this right only, to be the executive heads over 350 officers ;—and thirdly, I shall state the actual condition of the body of the Service intrinsically, and as compared to the army to which it belongs, as well as to the corresponding department of Her Majesty's Army.

1. One of the first general disadvantages of the Medical Department, as compared to the other services, is, that neither at home nor abroad is the interest of the Service represented in the councils of the State. In this respect it remains just what it was sixty or seventy years ago, although the general importance of the department and the qualifications of its members have increased enormously :—it is in the scale of remuneration alone that a great decrease has taken place. The Medical Service of India claims the consideration of Authority, on the score of its skill, labour, and activity, which have never been confined to mere recognition of professional duty, but extended to the developing the resources of the Empire at large, and to the moral improvement of its vast population. In these respects no class of the Company's servants has a higher claim to the consideration of the ruling power.

2. During the last sixty years the formation, discipline, and economy of the Indian Army have been variously and advantageously modelled upon those improvements, the results of experience, which have been adopted in the Royal Service; whereas the Indian Medical Department, an intrinsic part of the Army, has, by a strange oversight, been left just where that of Her Majesty's Service was in 1780 :—in other words, it has remained stationary during these sixty years ; and up to this hour it is deemed to have no claim to any other mark of distinction than is included in the mere routine of promotion by seniority :—in fact, the stagnation of this anomalous principle weighs upon it with peculiar and deadening effect—especially in Bengal, where promotion is so far more slow than at the other Presidencies. The great body of the Service—debarred the legitimate expectation and hope of rise or distinction, beyond such as belongs to a name borne so many years on the muster-roll—rather than to a character for knowledge, activity, and discernment—droops and stagnates—so as, after a time, to lose that salutary zeal which prompts to those exertions that, in all other services, secure a just and honourable reward.

3. Disadvantages press on every grade of the Service : they comprise—exclusion from the honors and privileges attached to military advancement, though medical officers be equally exposed with their military brethren to the most pressing dangers of Indian Service, as evidenced by the numbers that fell in the Burmese and Affghan campaigns, as well as in the earlier campaigns of Lord Cornwallis—a low military rank—a too low rate of retiring pension, as well as the want of a greater number of grades; in short, the entire Service demands a remodelling, to keep pace with the general improvement of the Indian Army, and to assimilate it to the medical service of Her Majesty's Army.

4. Many of the evils of the administration of affairs in general, in India, are traceable to a disposition to multiply checks so as in reality to cumber the executive machinery, thus casting the responsibility, not upon qualified individuals, but upon boards. In the medical department a board, instituted for the transaction of public business, consists, like the former board of the British army, of "three persons of equal power and authority; and as it rarely happens that three men think exactly alike in every thing, so counterpoises existing, counteraction arises, movement is jostled, embarrassed—and effect is feeble or erroneous"—in fact, a board so constituted presents us with the very symbol of *vis inertiæ*.

Judging by an extensive observation of more than twenty years, I should say, that of all the modes of perplexing simplicity of operation, of weakening energy, of nullifying unity of purpose, of clogging public business, and of evading substantial responsibility, a board is the most direct mode; but especially a board constituted on the seniority principle.

5. In Bengal, the members of the board have, on an average, completed a period of forty years' service. They are necessarily superannuated, generally deficient in military experience, and therefore cannot be expected to represent the feelings or the interests of the body of which the board is the nominal head: as members of a scientific corps, likewise, they represent an age gone by. Nor does the board possess the confidence of the Service, or of the Government, for power and patronage are taken away from it; thus leading to the conclusion that the Government of India does not consider the board to possess those claims founded on distinguished services and talents, large acquirements, liberal and generous views, conjoined to official tact, which ought to characterise the heads of a great department.

6. With reference to this part of the subject, a document lies before me, entitled to great consideration, as the testimony of a gentleman of distinguished talent and rank in the Civil Service; I mean Mr. R. D. Mangles, at present M.P. for Guildford, who, in his minute as member of a committee appointed by Lord Auckland, after declaring his opinion that no means short of a complete re-organization of the whole economy of the Bengal Medical Service can ensure efficiency, observes as follows:—

"It is impossible not to remark, in the first place, the utter want of professional stimulus to those exertions by which the other branches of the public service, the individual and the state, are alike benefitted. The existence of valuable prizes, which are happily, in many instances, awarded to merit, calls out from the ranks of the civil service the individuals from whom the local government, or the authorities in England, no less for their own credit than in justice to desert, select their highest and most distinguished functionaries; and many, doubtless, are quickened to highly useful efforts after distinction by aspirations for rewards which they never attain.

"Moreover, the recompense of success, almost always consists in the elevation of the meritorious individual to a sphere of more arduous labour and of greater responsibility in his own particular walk of the service, and invariably within its general pale, with the attendant benefits of enhanced consideration and emolument. A correspondiug state of things exists, though with some exceptions and in a qualified degree, in the Military Service; and those most competent to form an opinion on the subject, have always deeply regretted the points of difference, especially the want of a sufficient number of strictly professional prizes—a want which—as an apt example, compelled the Government of the day to reward the officer who so greatly distinguished himself at Seetabuldee, with a paymastership."

"But in the Medical Department there are scarcely any professional prizes proposed to merit.

"Under such a system, where every situation of pecuniary value is given upon a sort of tontine principle, as a bonus on longevity, and where, consequently, such situations are unattainable, out of turn, by the highest merit, and confer no distinction when attained; how is it possible that emulation, the mainspring of all useful and honourable exertion, should exist? How, again, is it possible," continues Mr. Mangles, "that the three senior surgeons on the list should always be properly qualified to instruct, direct, and control the whole body of subordinate officers; how the ten following names should always represent that number of efficient superintending surgeons? How would the affairs of the Revenue Department, to take an example out of many, be administered, if the board were invariably composed of the three senior members of the service, and the ten next on the list had a claim of right to the commissioners?" To Mr. Mangles' able and apt illustration, nothing need be added; for, by the seniority system, no one can be said to rise:—it is indeed, on the contrary, a steady progress downward in all that constitutes professional character. I leave this part of the subject then, only calling attention to it on the score of its high importance, and proceed to notice briefly the condition of the Service at large.

7. The Medical Service of Bengal consists of 350 officers. The proper training of such an extensive department to the efficient purposes of the army, requires a careful and systematic organization; for without that, and a competent individual at the head of the department, (or more properly speaking, as its responsible head), no talent or skill of the members of the general body, acting on no common system, and stimulated by no steadily succeeding vivifying motives, can avail for the public good. As it is now, a languid or cold indifference takes place of the better feelings that lie dormant in the Service.

8. The great improvement in the medical department of the British Army, and consequent amelioration of the soldier's health, date from the modification of it by substituting a director-general for a decayed board; and a regular gradation of officers of high rank and advanced allowances promoted from the more effective of the regimental surgeons. It is to this systematic arrangement under an efficient head, together with the honors, distinctions, and rewards distributed to talented and meritorious staff officers, that the present pre-eminent efficiency of the Medical Department of Her Majesty's Army is due. The Medical Department of the Indian Army should, without loss of time, be formed on the same model as that of Her Majesty's Service, only granting a higher rate of Indian pay and retiring pension to such regimental surgeons as should be found ineligible for staff employ after an actual service of twenty-five years. Such a measure would become necessary as a means of inducing persons to retire from active duty, and make way for more able successors. No arrangement that does not comprise a complete re-organization of the Indian Medical Department, can lead to or secure its real efficiency.

9. It arises from the general defects in our medical administration, and consequent want of emulation in the Service at large, that no one instance of honorary distinction or reward of any kind is to be found in a list of 350 medical officers, while it is notorious that there have always been numbers distinguished for professional merit, and many more who have shared the worst dangers and hardships incident to active Indian military service. The condition in this respect, of the Bengal Medical Staff, is most humiliating, and will of itself go far to account for the otherwise inexplicable fact of so very few, comparatively, out of so large a number, having risen to public distinction:—the talent is ample, but the prize for exertion has never been held forth.

10. There are now in the Bengal Army 112 officers promoted by Her Majesty's Brevet, and 49 officers of various grades on whom different orders and honorary distinctions have been bestowed, while the number on whom staff-offices have been conferred is scarcely to be reckoned. The difference here pointed out will appear more remarkable when it is considered, that the training of the military surgeon is effected in much the same manner as that of any other military officer; that is, after receiving a good general and professional education, he must serve with the troops in the field and cantonments, so as to acquire that practical experience, without which the best instructions of schools, or of civil practice, will yet leave him comparatively useless. It is notorious besides, that the sufferings of the Medical Staff, from the exposures and hardships incident to service in the field, even exceed those of the infantry.

11. Every officer of the Indian army, excepting those of the Medical Department, has it in his power to retire after certain periods of service on a pension equal to the full pay of officers of similar grade and rank in the royal army. Surgeons alone form the exception to the rule; for they are limited, whether they have served 20 or 32 years, to a pension of 10s. 6d. a day, while, as is shewn by the subjoined Table, the Surgeon in the Royal Army enjoys a graduated scale of retirement varying from a minimum of 10s. a day, after 20 years' service, to a maximum of 17s. a day after 30 years' service, with the prospect of a still higher rate if fortunate enough to attain the rank of Inspector or Deputy-Inspector of Hospitals. It thus appears, that the Indian Medical Establishments do not enjoy the improvements in pay and constitution of their department, in all its parts, that have been from time to time adopted in the Royal Army, upon which the Indian Army is modelled: they remain in point of rank and pension just what that of the Royal Army was prior to 1780. This is believed to be a pure oversight, as it never could have been intended by the Honourable the Court of Directors, the constitution of whose Military Establishments has uniformly been based on a perfect equality in rank and pension with the Royal Army, to leave so large and meritorious a department as the Medical Service of India, consisting of 720 officers, in so disadvantageous a position in this respect. Nor does the medical service of India bear any approach to that of the royal army in the number or importance of its staff offices; and great numbers in the Royal Medical Service are distinguished by British and Foreign honorary orders, whilst out of the Indian Medical Staff, consisting of 720 officers, not one instance of any such distinction is to be found. These facts are the more surprising when the very severe nature of Indian Military Service,* and the merits of the Medical Staff are considered.

12. There are only four medical grades in the Company's Service, viz. the assistant surgeon ranking with a lieutenant, the surgeon with a captain, the superintending surgeon with a lieutenant-colonel, and the member of the medical board with a colonel. We have no grade equivalent to that of major, which has been for many years a subject of complaint from its manifest injustice in a variety of ways, but hitherto the memorials on the subject have pro-

---

* As an example of the comparative severity of Indian service; the writer of this report begs to state that he served in the two campaigns in Ava, in the first year of which alone 3½ per cent. were killed in action, and 45 per cent. perished by disease—making a total loss of 48½ per cent.; whereas, taking a period of 41 months, during which the Peninsular War was prosecuted with the utmost vigour, 4 per cent. were killed in action, and 12 per cent. died of disease —"consequently each person employed throughout that year encountered more risk of life than in three Peninsular campaigns." In the second year of the war the proportion of deaths in action and by disease was "about one-half of what occurred in the first"—making the total loss, during the two campaigns, about 72½ per cent. of the European force engaged.—*Major Tulloch's Statistical Reports, printed by order of Parliament.*

duced no effect. The following are the grades in Her Majesty's Army, with the rates of pay attached :—

| | After 20 yrs. Service. | After 25 yrs. Service. | After 30 yrs. Service. |
|---|---|---|---|
| | per annum. | | |
| Director General .. .. .. .. .. | £2000. | | |
| | per day. | per day. | per day. |
| Inspector General of Hospitals .. | 20*s*. | 25*s*. | 30*s*. |
| Deputy Inspect. Gen. of Hospitals.. | 14*s*. | 17*s*. | 20*s*. |
| Staff Surgeon, 1st Class.. .. .. .. | 12*s*. 6*d*. | 15*s*. | 17*s*. |
| Staff Surgeon, 2nd Class .. .. | 11*s*. | 13s. | 15*s*. |
| Regimental Surgeon .. .. .. .. | 11*s*. | 13*s*. | 15*s*. |
| Assistant Surgeon .. .. .. .. .. | 6*s*. | 7*s*. | 7*s*. 6*d*. |

N.B. By the pension-rules of Her Majesty's Service, 20 years' passed in the East Indies are equivalent to 30 passed in the other British dominions.

In the present condition of the Indian Medical department—deprived of grades and honors—an officer, when he has attained the rank of surgeon, may consider that for the next twenty years he has nothing further to hope, and that promotion will reach him only with superannuation. This state of hopelessness causes many to retire from the service at a time of life when they are most useful to the State.

13. On attaining the long-envied rank of superintending surgeon again, the incumbent must serve two years before he can lay claim to the pension of his rank; whereas the military officer has only to be Gazetted to possess his right of pension for life, from the date of such Gazetting. This is considered a particular hardship in the medical service, and with justice it is believed, when we see that at present the ten senior surgeons on the Bengal list are of 30 years' service, or upwards, who, according to existing rules, are entitled to no higher rank or pension than that of captain, viz. £191. per annum. In the Royal Army the rate of pay progresses according to the date of standing in the Service.

"All English labour in India," it has been well observed, "from the labour of the Governor-General and Commander-in-Chief, down to that of a groom or watch-maker, must be paid for at a higher rate than at home. No man will be banished, and banished to the torrid zone, for nothing." This rule seems admitted in respect to both the pay and pension of all public functionaries in India, with exception only of the Medical Department of the Army.

14. Let the following facts speak for themselves :—a captain retires on completing 24 years' actual service in India, on major's pension, or £292;—a captain of 28 years' service, on lieutenant-colonel's pension, or £365;—a captain of 32 years' service, on colonel's pension, or £456;—while a surgeon of 24, 28, and 32 years, has yet but a pension of £190, for service solely in a tropical climate; and we have now, in Bengal, surgeons of 33 years' standing, unpromoted. Again :—one third of the colonels of the army have regiments, affording a competency for life; and of 191 lieutenant-colonels and majors, one in two is in receipt of extra-regimental allowance for the command of corps. Of the field officers besides, 56, or one in five, hold staff situations with handsome salaries; and of the captains, 159, or one in three, hold similar advantages, while 389 lieutenants, or one in 2½, hold staff offices. In addition to all these advantages, the officers of the army have the privilege of purchasing out seniors to an unlimited extent. Now, of 106 surgeons in Bengal, only ten hold staff appointments with allowances above regimental surgeons; and of 244 assistant-surgeons, 14, or one in 18 only, have the same advantage, while in the army of the same presidency, one officer in six holds field rank, and only one surgeon in twenty-six stands in corresponding position. The comparative disadvantages under which we labour, are as numerous as they are impolitic in themselves, and humiliating to our service.

15. It is important here to notice the economic fact that, with the double advantage for retiring possessed by the army, the home pension list has not increased of late years. It is clear, therefore, that the granting of similar advantages to the medical department would not entail any additional cost: on the contrary, by removing the superannuated, who draw the larger salaries in India, the efficiency of the service would be promoted, and that without adding to the home expenses of the Government.

16. If, as here taken for an undisputed fact, the principal object of the Government is, and ought to be, the formation and maintenance of an efficient Medical Staff for the army; then the existence of the various civil abuses already pointed out, and the employment of so large a portion of the service for an indefinite term of years in civil stations, are most injurious. The engrossing commercial habits acquired at civil stations, and not unfrequently, the habits of professional inaction, induced by want of practice, tend greatly to disqualify such as have passed a number of years in such a situation for a return to their proper duties with the army. This most injurious course should instantly be put a stop to, and no surgeon or assistant-surgeon ought to be permitted to remain longer than three years in civil employment of any kind, except at large city stations, where there are hospitals; and such as must of necessity be so employed should be encouraged to practise amongst the people, and to study the medical topography of the districts in which they serve, so as to keep their minds in professional exercise. The much abused custom now prevalent, of permitting medical officers to resign promotion, holding for life civil and military staff offices of responsibility, ought also at once to be abolished, as being injurious to the character and best interests of the service.

17. Promotion to offices of trust should no longer be granted on the seniority principle. To demonstrate the truth of this cannot be necessary, after what has been already stated; but I may as well mention that, in Bengal, an individual of little worth, whether regarded as a medical officer or a gentleman, may now look upon it as certain that, provided he survive his contemporaries, he shall rise in due time, not only to the superior staff employs, but to the governing head of the service; and thus have risen within the writer's recollection, not once, but frequently, the mere merchant, the confirmed gambler, and the exhausted tippler It is true that in our motley and vague code of regulations the principle of selection is paraded forth; but in effect it has remained a dead letter—a mere mockery.

The depressing influence of such a system, is not to be told; and it ought to be henceforth a rule of the Service that no officer but one of the highest character, moral and professional, should ever rise to such offices; and experience, with approved service with the troops, both in the field and in cantonment, ought to be a qualification in no instance to be dispensed with.

To conclude: As respects the existing state of the Bengal Medical Service, I think it has been amply made out, that with reference to its directing head, the Staff immediately subordinate to that head, and the general distribution of the members at large, a more defective department exists not in any army. Under such a system of misapplication of means to an important end, the talents, the emulation, the aspiring feelings, no less than the experience even of the Service, are paralysed, and will continue to be so, until a more healthy and vigorous plan of supervision and general organization be established.

J. R. MARTIN.

*London, June* 10, 1840.

---

### TO RECAPITULATE.

1st.—That pension, according to length of service, be extended to the Medical Service of India, the same as granted to the officers of the Indian army, and as accorded by the British Government to the medical branch of the royal army; also, that the superior medical officers be entitled to their pensions from the date of their respective commissions, in common with the military officers in India, and with the medical officers of Her Majesty's service.

2nd.—That honors and rewards, proportionate to its services and experience in actual war, be awarded to the Indian Medical Department, and such as are granted to the medical branch of the royal army.

3rd.—That grades similar to those of the royal service, be established in the Medical Department of India, and that the Governments be no longer limited in their selection for responsible staff and other medical offices to mere seniority; but that, after the prescribed period of seventeen years' service, all officers of character, and who may have served with credit with troops in the field, be eligible to the higher staff and other employments; professional character and military experience in actual service, being indispensable.

4th.—That no medical officer of whatever rank be allowed to continue longer than three years at a civil station, or in civil employment of any kind, with the exception of large cities, as already mentioned.

5th.—That the very injurious custom hitherto prevalent, of permitting officers to resign the active branch of the service, still holding for life staff and other responsible offices, be discontinued; and that, for the future, all such as desire to abandon the active duties of the profession be struck off the effective list, and transferred to the Invalid Establishment. J. R. M.

### ADDITIONAL NOTE.

1st.—Though the system of promotion by absolute rule of seniority, as it now holds in India, is without a defender either here or there, so far as I know; yet, as some few persons whose opinions I hold in respect, apprehend improper supersession, favouritism, and certain supposed abuses of patronage from the introduction of the plan proposed by me, I think it due to these opinions to state my reasons for non-concurrence in them.

2nd.—To combat the apprehensions here stated, I shall adduce facts, an extended experience, and comparison—all referring directly to the condition past and present of the Medical department of Her Majesty's Service; but before doing so, I would here observe, once for all, on the strangeness of the notion that three men, each having his friends and favourites to advance, and being screened from all responsibility by their very plurality, should be less prone to this apprehended abuse of patronage than one responsible head, "openly and solely responsible"*—having the eyes of the profession and of the public fixed upon him—a supposition opposed alike to principle and to actual experience.

3rd.—Unhappily for the interests of the Royal Medical Service, its affairs

---

* In utter hopelessness of efficient or responsible service from the then existing Army Medical Board, His Majesty George III. was advised in 1798 to constitute another Board—still another Board!!—consisting of three men, a were for a long time administered by a Board, nearly similar to those now existing in India; and the results were more calamitous in proportion as power and patronage were entrusted to the British Board.

These results were—an utter misapplication of all means tending to the welfare of the army and of the medical department—the horrible institution of the system of general hospitals, destructive of military life—an expenditure so wasteful as to defy belief, were it not for the direct nature of the proofs—a deterioration in the character of the service, inducing every one who could to leave it—all leading to the mismanagement of the soldier's health, and the consequent failure of national enterprises.

These were some of the leading effects, but not all, resulting from the institution of a Board in the Royal Service. They are now matters of history, and I may without another word appeal to the history of the working of the now existing system in Her Majesty's Service in proof, were any wanting, of its vast superiority as regards every object of military welfare for which a medical establishment is instituted.

4th.—I come now more particularly to the apprehended abuse of patronage; and here again I would meet opinion by "facts, an extended experience, and comparison," all furnished to us within the limits of our Indian Empire. For twenty years the affairs of the Medical department of Her Majesty's Army in India have, fortunately for that service, been directed by officers of the Royal Medical Establishment.

I say "fortunately," because they have without a single exception been men distinguished for character, talent and experience. Here then we perceive the British Military Authorities and the Director General, neglecting this fairest and richest of their fields for abuse of patronage, in favor of unpretending but real merit; and those who have visited the Colonies of Mauritius, Cape-of-Good-Hope and Australia, have found in them the same honest regard to long and meritorious services. These are well-known facts in the experience of the last twenty years; and I leave to others the melancholy and ungracious task of comparing them to the working of our Indian Boards during the same time.

5th.—In the plan proposed by me, the utmost weight is given to long service when joined to character; in other words, always to select the older when the fitter officer; but as in no walk of public life are all men fit for promotion to the higher departments, the pension according to length of service is claimed for medical officers in common with their military brethren.

6th.—To conclude:—Were it as true in nature, as unhappily it is the reverse, that the best men live the longest, still the system of rise by the muster-roll would be the very worst:—repressive of all emulation in youth and manhood, and deferring promotion till the verge of life, it leaves for the discharge of public duty no energy, moral or physical, in the possessors of high office.

Such would be the law of nature in any climate; but it holds especially in tropical regions, wherein the course of life is notoriously precocious of old age.

J. R. MARTIN

*Grosvenor Street, Grosvenor Square, London,*
*October 25th,* 1841.

---

Physician-General, Surgeon-General, and Inspector-General, having each "his distinct province of business," and to be held "openly and solely responsible for his own acts." How vain was this last attempt of the British authorities is now matter of history; and every day's experience proves that, however respectable individual members may be, "the moment men meet in public boards they cease to be collectively excellent."—*Vide Fifth Report of The Commissioners of Military Inquiry—Army Medical Department.*

To Sir WILLIAM BURNETT, K.C.H. &c. &c.

*Royal Hospital at Haslar, 8th August,* 1842.

Sir,—John Williams, private, Royal Sappers and Miners, aged 26, of great strength and activity, but addicted to habits of intemperance, has been employed during the last two summers on the wreck of the Royal George at Spithead, as a diver, and is considered one of the most expert workmen. On the morning of the 11th ult., clothed in his submarine armour, he was engaged at the bottom of the sea, at a depth of 80 feet from its surface, in fastening an iron chain round a block of wood, that was imbedded in the stiff mud; which task, after an hour's labour, he had just completed, when the flexible tube that supplied him with air suddenly burst above water, with a loud hissing noise, which was distinctly heard at the distance of fifty fathoms.

While the divers are employed in their laborious search under water, the utmost vigilance is exercised by those on the deck of the hulk, to prevent or remedy anything that might endanger the diver's life, or interrupt his operations. The persons, accordingly, who were stationed at the air-tube, and life-line, by which the divers are assisted in their ascent, immediately perceived the accident that had happened, and one of them closed the hole in the tube with his hand. Williams was promptly hauled up, but his armour got entangled in the heavy rope-ladder, by which the divers descend, and he and it were pulled up together, in the space of about a minute and a half, from the occurrence of the accident.

On removing the helmet from his head, blood was seen running in a stream from his ears, nose, and mouth. His face and neck were swollen and discoloured; he looked faint, but was sensible. In this state he was conveyed to the hospital, where he arrived in an hour after the accident. His face then was one mass of lividity; his neck was excessively swollen, bloated, and suffused with livid coloured blood. Dark patches of ecchymosis that did not coalesce existed over the clavicle and shoulders, with intervening spaces of skin of the natural colour. The lower part of the neck, which had been covered with the flannel and India-rubber dress, was mottled black and white; the dark ecchymosis being raised in lines, with slight streaks of white skin interposed. The livid discoloration of the face extended upwards to, but did not pervade the hairy scalp, where it terminated abruptly; nor were any spots seen below that part of the chest which was covered by the helmet. The lining membrane of the cheeks, under the tongue, over the fauces and pharynx, as far as the eye could reach, but especially over the tonsils, was black with ecchymosis. The conjunctivæ where they are uncovered by the eyelids, and particularly round the margin of the corneæ, were turgid with black blood. He vomited some blood before he reached the hospital, and he afterwards made occasional efforts to vomit, apparently from the accumulation of blood in the fauces, which blood he now and then expectorated. The hæmorrhage had ceased from the nose and ears, which were still covered with clotted blood. He was perfectly sensible, but seemed drowsy; pulse 76, of natural strength; breathing interrupted by frequent, deep, involuntary sighs.

Lieut. Hutchinson, who was present when the accident happened, and who accompanied Williams to the hospital, said that the swelling of the face and neck had much increased, and the lividity had much deepened, during the hour that had elapsed since he left the hulk.

In the course of the same day, the lividity of the nose and point of the chin vanished, and those parts resumed their natural colour. The colour of the face too became much paler, in proportion as the vessels recovered their freedom and diameter; but there were large patches of extravasated blood in the eyes, mouth, face and neck, which could only be removed by the tedious process of absorption. On his admission, warmth was applied to his extremities; some warm tea was given him, which he swallowed with the greatest difficulty; he had a turpentine enema; and in the course of the day twenty ounces of blood were taken from the arm. The following morning a senna draught was prescribed, which has been occasionally given since. He has complained of occasional headache and dimness of sight, from which he is now free. The swelling and ecchymosis of the face and neck have daily diminished; and these parts have now attained their natural size and colour, showing that they were swollen, on his admission, to twice their natural size. The ecchymoses under the conjunctivæ were very tardily absorbed, and minute clots were visible for three weeks around the union of the cornea and sclerotic coat.

A similar accident occurred, under the same circumstances, about twelve months ago, to Private Roderick Cameron, of the same corps, whose head, neck, and eyes were discoloured in the same way; an account of which has been read to the British Association by Dr. Richardson. When Cameron was hauled up on the hulk's deck, he had lost all consciousness, and was in a state of apparent asphyxia, from which he soon recovered. A little blood only escaped from his nose; and none from his ears or mouth. At the expiration of a month, the ecchymoses under the conjunctivæ, that remained the longest, had disappeared; and undaunted by the perilous accident which had jeopardized his life, he returned to his work as a diver, which occupation he still fearlessly follows. Williams too is undismayed by his frightful accident, and he has resolutely returned to pursue his adventurous life; feeling confident that the prudent application of those precautions which are now well known, will secure him from danger.

An accident somewhat analogous, as far as I can make out the facts, occurred to a diver at the wreck of H.M.S. Thetes, in South America, who, with a companion, had descended in a diving-bell. In this case, also, the air-tube burst and one of the men immediately extricated himself from the diving-bell, and rose to the surface of the water unhurt. The other man, by some means, got entangled, and was some time in freeing himself from the bell, which he at length accomplished by the aid of his companion, who again descended for the purpose. When he reached the surface of the water, he was much exhausted, and his face and body were blackened with ecchymosis down to the *waist*. This discoloration gradually went off in the course of a month, the blackness of the balls of the eyes being the last to disappear.

These curious and strikingly similar effects of the same kind of accident at Spithead seem to arise from the sudden removal of the compressed air, and the consequent exertion of the pressure of the superincumbent water on those parts of the body which are not covered by the unyielding helmet. It is calculated, in round numbers, that the pressure of the water on Williams' body, at the depth of his submersion, at the time of the accident, was nearly equal to the weight of three atmospheres, which pressure was counteracted, and the equilibrium preserved, by throwing air through a forcing-pump, of great power, along a flexible tube into his helmet. This supply of air is steadily kept up, by constant regulated pumping, during the whole time the diver is under water; and until the centre lens of the helmet is opened on deck. The quantity of air thrown along the tube into the helmet, far exceeds what is required to sustain easy respiration and the equilibrium of pressure; but no harm can result from any additional quantity of air that may be forced in; inasmuch as the superfluous air readily escapes into the sea, by a valve in the helmet, and is seen constantly bubbling up on the surface of the water. When the tube burst, and the air escaped from the helmet, this equilibrium of pressure and resistance was destroyed. The head was protected by the strong helmet, which did not collapse, from the pressure of the circumambient water, which now acted on the rest of the body with a force equal to two atmospheres, and produced a feeling, as he expressed it, as if he had been crushed to pieces by his dress. The blood thus driven from the extremities and from those parts of the body that were not covered by the helmet, was forced into the vessels of the head and neck (as it is into a part of the skin placed under a cupping-glass) some of which blood remained in the vessels and disappeared in a few hours after the accident; but a large portion was extravasated in the loose textures into which it had been forcibly driven.

Six divers have been employed, during the last three Summers, on the wreck of the Royal George. They have now nearly succeeded in clearing it away, and the anchorage has been, in part, restored to its former security, with fewer accidents, and none of them fatal, than it is likely would have occurred in similar operations, during the same space, above water. The value and importance of these operations and the extent to which they may be applied in recovering treasure, or in the destruction of important works on an enemy's coast, are still imperfectly understood, but General Paisley has satisfactorily established that these sub-aqueous operations may be accomplished at any ordinary depth, with ease and safety, by men who are not professed divers, and who have not been trained up from their infancy to the art. The divers engaged at the Royal George have been selected from the corps of Royal Sappers and Miners, whose characters and abilities as steady men and good workmen were known to their officers; but only some of those so selected have become good divers, for the effects of protracted submersion are so different in different individuals that it is not every man who can follow the perilous life of a diver. Many experience intense pain in the ears and bleeding at the nose during their descent; and Lieut. Hutchinson, who ably conducts the operations on the Royal George, always experiences these sensations, and he has never been able to remain under water for any length of time. Those who are accustomed to dive successfully, never suffer any such inconvenience, and I cannot learn that they experience any very marked uneasy sensation, unless an occasional sense of nausea, or distention at the stomach, headache, and rheumatism; but they all agree that they are much weakened and wasted by the exertion, and as they express it, are not the men they were when they began the occupation. I have not had sufficient experience to determine whether it renders them permanently unhealthy or short-lived.

The diving season commences in May and ends in October, and the divers are usually employed eight or ten hours in the twenty-four. No scene can be more striking, than the activity that pervades the Hulk during the immersion of the divers. The busy groups of pumpers, on whose regulated exertions the lives of the divers depend; the deep groanings of the air-pumps; the anxious care that the men stationed over the hulk's side, with the air-hose and life-line in their hands, bestow on the preconcerted signals, by which the divers communicate their wants with the precision of speech; the turbulent agitation of active ebullition, that is occasioned by the forced escape of the compressed air through the sea; the dreadful plunge and rapid disappearance of the enormous and unwieldy mass in this boiling cauldron; the eager look of expectation and vague apprehension, with which the spectators gaze on the whole process, exceed in interest any thing I have ever witnessed.

The divers remain under water, according to the nature of their work, from half an hour to three hours; and although, in order to accelerate their descent, they are heavily laden, with ponderous shoes and large leaden weights on their shoulders, constituting a dress of a hundred and thirty pounds weight, they move about nimbly at the bottom of the sea, and feel and work as lightly as if they had nothing on their shoulders and feet.

Mr. Richard Tilston has kindly made a very correct drawing of Williams's appearance when he was brought to the hospital; to which he has added a faithful picture of him, in his working dress, at the time the accident occurred, in order to convey a notion of his mode of groping his way under water, with his pricker in one hand and dog in the other. In these sub-marine expeditions, the divers frequently encounter each other. On one occasion, three of them, from two different hulks, met, joined hands in a circle, and gave three hearty cheers at this triumph of the art of diving. On other occasions, however, their meetings are less friendly; disputes arise as to their claims to particular logs of wood, quarrels and sparring ensue, in which their large iron prickers play the part of single-sticks.

Nothing can exceed the spirit and industry with which these operations have been conducted, or the laudable emulation displayed by the workmen to excel each other in the quantity of work they severally perform ; and nothing has occurred to damp their ardour until lately, when Corporal Jones, one of the most courageous and useful divers met at the foot of his ladder a dead body, which produced such a shock, that he immediately ascended in the greatest possible alarm ; and spoke as if he had encountered a supernatural being. Unsuccessful attempts were made to rake the body up from below ; but a few nights afterwards it was brought up by Corporal Harris, the most intelligent and persevering of the corps, on his pricker, without his knowing what he had got hold of. His consternation when he reached the surface and found that it was a dead body, was so great, that he could not go down again, and he was replaced by another diver, who not having felt his horror, was not unwilling to descend.

The divers are employed four hours at a time, during the slack tide of low water, and in that space they usually descend about four times. On their ascent after an hour's submersion, they appeared to me, while they were leaning against the hulk's side, to be pale, languid and exhausted, though they did not admit that they were fatigued. When they reach the top of the ladder, the centre lens is unscrewed, their ponderous helmet is taken off, and they are generally allowed ten minutes to recruit, while the wood they may have collected is drawn up by a crane. They can only work two hours at the slack tide of high water in consequence of the strength with which the tide ebbs and flows at that period, which, they say, begins earlier and runs with much more rapidity at the bottom than at the surface of the sea, and which would carry them off their legs. They are also sometimes interrupted by storms, that would prevent their signals from being understood by those who attend the air-pipes and life-lines on the hulk's deck.

The divers are clothed in flannel dresses, that fit closely, which retain the warmth of the body and prevent the chill that might be produced by the soaking of the water through the seams of the India rubber dress. This dress is protected on the outside by a canvass covering, from any injury it might sustain by rubbing against the nails or ragged pieces of the wreck.

We know little of the effects produced by the respiration of compressed air, but the divers find that they can breathe easily at the bottom of the sea ; they can sing readily, but cannot whistle. They converse with each other, by shouting at the top of their voice, which they hear in a whisper.

Each diver is paid, besides his regimental day pay of one shilling and threepence, two shillings a tide, working three tides in the twenty-four hours.

Since the occurrence of Williams's accident, each diver has been furnished with a safety-valve, placed between the end of the air-pipe and the helmet. The air being forced in from the pump, opens the valve, and passes into the dress, but the moment this pressure is removed, the valve closes and prevents any air in the helmet returning through the pipe. This contrivance, however, I fear, in the event of an accident, would only substitute suffocation for that of being squeezed to death.

I have the honor to be, Sir, your most obedient servant,

JOHN LIDDELL.

